# Virus Removal from Real Wastewater as an Environmental Management Approach

**DOI:** 10.3390/molecules29235601

**Published:** 2024-11-27

**Authors:** João Gomes, Eva Domingues, Danilo Frasson, Rui C. Martins, Ana Miguel Matos

**Affiliations:** 1 CERES, Department of Chemical Engineering, Faculty of Sciences and Technology, University of Coimbra, Rua Sílvio Lima, 3030-790 Coimbra, Portugal; evadomingues@eq.uc.pt (E.D.); danilobr_mf@hotmail.com (D.F.); martins@eq.uc.pt (R.C.M.); 2 CERES, Microbiology Laboratory, Faculty of Pharmacy, University of Coimbra, Pólo das Ciências da Saúde, 3000-548 Coimbra, Portugal; anamatos@ci.uc.pt

**Keywords:** pest management, *Corbicula fluminea*, biofiltration, JC virus, norovirus, hepatitis A virus

## Abstract

The increased presence of resistant microorganisms in water promotes the need for supplementary measures to mitigate the water source’s contamination. Traditional treatments are inefficient in wastewater management at removing some emerging contaminants. *Corbicula fluminea,* an invasive species, can be used in the treatment due to their resistance and biofiltration capacity, working as a pest management strategy. In this study, this bivalve was used to promote the virus disinfection from the municipal wastewater treatment plant (MWTP) that enters (influent) and after the secondary treatment (effluent leaving the plant). JC virus, norovirus (GI, GII), and hepatitis A (HAV) were identified. *C. fluminea* promoted norovirus GI and GII removal after 72 h and a slight decrease in the JC virus concentration. These results prove the potential of this pest management approach to be used in virus removal. Furthermore, infectivity assays using mengovirus confirmed the correlation between the presence of the genome detected by PCR and the infectious virus particles. This highlights the potential of PCR as a reliable indicator of the infectious virus’s presence. However, such an infectivity assay proved that even when PCR results are undetectable, a reduced number of viruses may remain viable and able to infect susceptible cells in culture.

## 1. Introduction

Over the last few decades, due to the degradation of natural environments, the constant growth of the population and industrial sector, and the usage of various chemical compounds to increase productivity and quality of life, different environmental problems have been reported around the world [1]. Water scarcity is rising, and it is expected that by 2030, 47% of the world’s population will live in areas where water supply cannot meet the demand [2]. Therefore, it is important to find alternative water sources, such as wastewater reclamation and water reuse strategies [3]. However, conventional treatments cannot face refractory chemical compounds as well as biological emerging compounds such as enteric pathogens [4]. In recent decades, many new pathogens such as bacteria, viruses, and protozoa have resulted in water-borne disease epidemics, mainly due to their presence in the wastewater in great extension [4,5,6,7]. The most common bacterial indicator of fecal pollution is *Escherichia coli* and is included as a parameter control [8] for wastewater reuse. However, the absence of a correlation between the levels of bacterial indicators and viruses in treated wastewater has been reported. Thus, conventional indicators may not be appropriate for evaluating treatment efficacy for human virus removal [9], and the use of some human enteric viruses as a fecal contamination indicator has also been applied in effluent quality tests [5,10].

Enteric viruses are widely present in treated wastewater, which may be detrimental to human health if contact is established. These enteric viruses are listed as emerging contaminants on the United States Environmental Protection Agency Contaminant Candidate List [11] in drinking water treatment. However, there is still no regulation to monitor their concentration in treated wastewater discharges [5]. When in direct contact with individuals via the fecal–oral route, or indirectly through food or environmental contamination, these enteric viruses can cause infections such as gastroenteritis, conjunctivitis, and respiratory problems [5]. Different viruses, such as hepatitis E virus, human adenoviruses, and JC and BK polyomavirus, which are not normally monitored in terms of waterborne diseases, have been evaluated in samples taken from river water and sewage. The efficiency of the drinking water treatment plant was also analyzed, and two log removals (99%) of JC polyomavirus and human adenoviruses were observed. Moreover, it was concluded that quantifying these viruses is useful for the quality index of source and treated water control [12].

The chemical disinfection procedure of the water supply is the main solution for preventing and reducing illness involving humans and drinking water usage [13]. The formation of by-products can bring even more health effects and is related to the reaction between the high doses and/or inappropriate disinfectant and other compounds in the wastewater [14]. Thus, there is a growing interest in alternative treatments in the absence of disinfectant by-products and their effective inactivation of microorganisms [15,16]. Some species contribute to the maintenance of freshwater system quality, such as clams and mussels [17], due to their biofiltration capability. New approaches involving environmental applications for these organisms, such as eutrophication control, pathogens, and contaminants biomonitoring and removal from water, are low-cost solutions and represent new methods to solve pest and environmental problems [16]. Freshwater clams live buried in sand, mud, and gravel in aquatic habitats [18]. Freshwater with oxygen and food is drawn into the inlet siphon, passing through two sets of gills where the gill cilia and mucus filter the particles/food and expel the deoxygenated water along with waste particles as pseudofeces [18,19,20]. Their natural filtering capacity includes the removal of contaminants and disease-causing organisms from water supplies [17,21]. The filtration rates of clams can be even higher than other filter feeders in the environment, filtering daily between 10 and 100% of the water column and presenting an uptake capacity of particles between 5 and 30,000 μm^2^ [22]. Different studies focusing on removing nutrients, organic compounds, metallic contaminants, and enteric pathogens have indicated that several environmental uses can be given to such invasive species. Selegean et al. [19] used invasive zebra mussels as a bacterial contamination indicator (*E. coli*) because of the high mussel’s biofiltration and accumulation capacity. Ismail et al. [23] used two different systems (batch and field-based flow-through) and a native species (*Anodonta californiensis*) to study the influence of initial concentrations of *E. coli* in the clearance rate. However, the relationship between the parameters was not linear, due to the turbidity of the lake water samples used in the batch system. Also, the use of native and invasive species, *A. californiensis* and *Corbicula fluminea*, in laboratory and situ-field studies were compared, and bivalve type and length are important parameters to be considered when bivalves are used as a biofilter [24]. Gomes et al. [16,25] also used *C. fluminea* for *E. coli* and organic matter removal from swine wastewater, and the number of clams per volume of wastewater is important to achieve better results.

In this work, water disinfection was studied using an invasive bivalve species, *C. fluminea*, as a filter feeder of enteric pathogens, analyzing their efficiency as an alternative pest management and their ability to remove human viruses commonly found in wastewaters. The bivalves were in contact with wastewater (influent and effluent) from MWTP, and the variation in viral loads was evaluated throughout the treatment process. The novelty of this work encompasses the application of clams at different stages of MWTP, which can help with the correct placement of bivalves for disinfection treatment. In addition, the spiking of samples with a culturable virus, such as mengovirus, enables the elucidation of the real efficacy of clam action in inactivating viruses present in wastewaters and correlates PCR results with the presence of infectious virus in this type of sample.

## 2. Materials and Methods

### 2.1. Water Sampling and Chemicals

**Figure 1:**
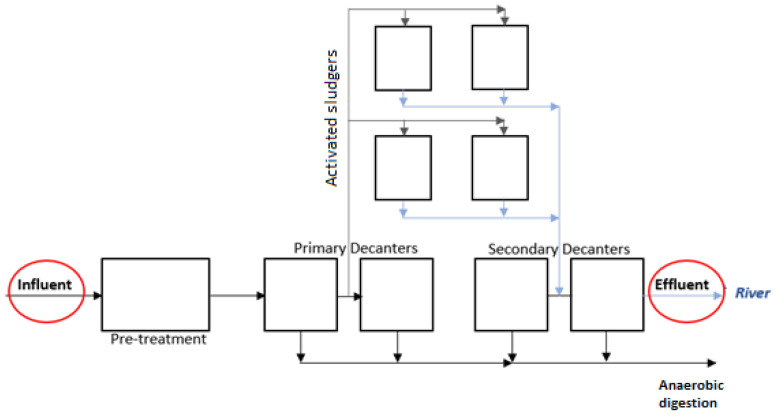
Scheme of wastewater treatment plant and sampling points.

The wastewater used in the experiment is provided from a municipal wastewater treatment plant, which treats wastewater with a flow rate of about 2400 m^3^/d. The plant is equipped with a primary settler, activated sludge treatment, and secondary settler, as can be seen in Figure 1. To study the biofiltration using *C. fluminea*, the municipal wastewater was collected at the entrance (before adding ferric chloride—influent) and at the exit after the secondary treatment (effluent) of that municipal wastewater treatment station was used (Figure 1). The influent and effluent sample locations are schematically indicated in Figure 1.

### 2.2. Experimental Conditions

The tested organisms, *C. fluminea*, were obtained from São Tomé’s canal located in Mira, Portugal (40°25′06.90″ N, 8°44′13.18″ W). The collection was carried out using a mesh bag to sieve the sediment; the clams with shell lengths between 16 and 27 mm were selected, properly stored with the canal water, and transported to the laboratory. In the laboratory, the bivalves were kept for one week at a constant temperature of 20 ± 2 °C, continuous aeration, and photoperiod of 16 h^Light^:8 h^Dark^. After that, once a week, the culture water was renewed with dechlorinated water, and the bivalves were fed with mashed spinach. This procedure is made over two weeks in order to remove the main pathogens that can be in the soft tissues of clams. One week before being used in the experiments, the organisms were placed in the dechlorinated water and not fed.

**Figure 2:**
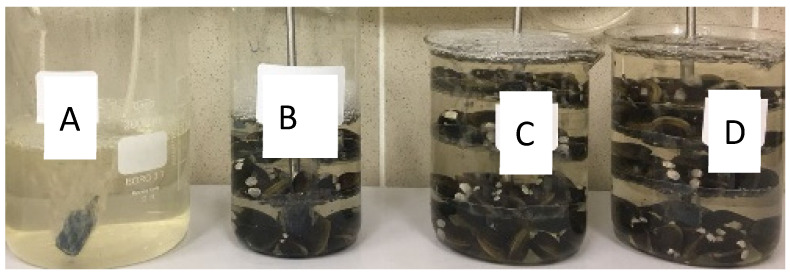
Experimental procedure for biofiltration experiments, (**A**) effluent without clams, (**B**) control of clam’s mortality, (**C**,**D**) replicates of biofiltration experiments.

Both wastewater samples (influent and effluent) used in the experiments were previously sieved (pore size of 5 µm) to remove the large debris and suspended solids before being placed in the biofilters. The biofilters composed of a beaker and the clam’s support (composed of three floors) were filled out with 60 clams and 1 L of wastewater sample (influent or effluent), which means a ratio of 1:60. This ratio was selected based on the previous work [16]. Each experiment was carried out with the respective blank control (wastewater sample without clams) and kept at a constant temperature (20 ± 2 °C) and continuous aeration. The biofiltration experiments with influent and effluent were made in duplicate. The standard deviation was lower than 2% for all experiments. Figure 2 represents the experimental configuration used in the biofiltration trials. Label A corresponds to the effluent sample without clams; label B is the control of clams’ mortality, which is made to validate the test since if the clams die, the test is not valid. In these experiments, no mortality was verified. Labels C and D are replicates of the biofiltration experiments.

To estimate the efficiency of the protocol for viral recovery, samples were spiked with mengovirus, strain MC_0_ (ATCC^®^ VR-1597), as a process control virus at the beginning of the experiment.

The biofiltration procedure using the effluent was carried out for 72 h, with 150 mL samples of each beaker (2 blanks and 2 biofilters) being taken at 0 h and 72 h for virus detection and quantification. Regarding the effluent sample, the biofiltration test was extended for 1 week (192 h) to verify if there is a significant reduction in JC virus. The procedure using the influent was also performed for 72 h, with samples for the virus test taken at the same points as the effluent experiment. The samples collected for virus quantification were immediately filtrated by a 0.45 μm cellulose membrane and stored in the freezer (−20 °C) until analysis. Samples taken for infectivity assays were immediately processed after collection.

Moreover, no virus adsorption onto the clam’s support was verified through the placement of wastewater samples in contact with clean support (without clams) for 72 h and 192 h.

### 2.3. Virus Analysis

#### 2.3.1. Virus Detection and Quantification

Virus detection and quantification throughout the experiments was accomplished by real-time polymerase chain reaction (qPCR). The procedure follows different steps, namely, virus concentration, nucleic acid extraction, amplification, and quantification of viral genomes. Viral particles in analyzed samples were concentrated through an ultracentrifugation protocol previously described [26]. Briefly, 75 mL of sample were ultracentrifugated (41,000 rpm) for 90 min at 18 °C for pellet formation. After centrifugation, the supernatant was rejected, and 500 µL was left to resuspend the pellet, to which 500 µL of chloroform was added. After homogenization and centrifugation at 1500 rpm for 10 min, the aqueous phase was collected and stored at −20 °C until nucleic acid extraction.

Nucleic acids were extracted from 140 µL of processed sample using the QIAmp^®^ Viral RNA Mini Kit (QIAGEN, Izasa, Carnaxide, Portugal) according to the manufacturer’s instructions. Viral genomes were eluted in 60 µL of elution buffer and stored at −20 °C until further analysis. Detection and quantification of viruses (both present or spiked in a wastewater sample) was performed on 7.5 µL of nucleic acid extract through qPCR protocols with primers and probes specific for each evaluated virus (Table 1), using a CFX96 Real-Time PCR Detection System (BioRad, Amadora, Portugal), briefly described below.

JC virus detection and quantification were accomplished through a qPCR protocol with primers and probes directed toward AgT and NCCR regions of the JC genome [27]. Briefly, 25 µL reaction volume containing 7.5 µL of nucleic acid and 17.5 µL of Maxima Probe qPCR Master Mix (2X) (Thermo Fisher Scientific, Porto Salvo, Portugal, K0232), with 300 nM of each primer, and 200 nM of TaqMan probe were submitted to thermal cycling protocol consisting of an initial 2 min incubation period at 50 °C, followed by 10 min at 95 °C, and 45 cycles of 95 °C for 15 s, and 60 °C for 60 s.

The evaluation of norovirus comprised the detection and quantification of genogroups I (GI) and II (GII) separately and was made by real-time reverse transcriptase (qRT)-PCR using specific primers and probes previously described [28]. HAV and mengovirus detection and quantification were also performed by qRT-PCR protocols with specific primers and probes previously described [29,30]. Detection of RNA viruses was accomplished with 7.5 µL of nucleic acid, added to 12.5 µL of SuperScript III RT reaction mix (Invitrogen, Amadora, Portugal 11732-020), 0.5 µL of SuperScript III RT/Taq Mix (Invitrogen 11732-020), 500 nM of forward primer, 900 nM of reverse primer, and 250 nM of TaqMan probe, in a final volume of 25 µL. The thermal cycling protocol consisted of an initial 1 h incubation period at 55 °C for reverse transcription, followed by 5 min at 95 °C, 45 cycles of 95 °C for 15 s, 60 °C for 60 s, and 65 °C for 60 s.

Estimation of viral recovery efficiency of the used process was accomplished through the analysis of spiked mengovirus. Briefly, 10^−1^, 10^−2^, and 10^−3^ dilutions of extracted mengovirus RNA in nuclease-free water were prepared. A standard curve resulting from the serial dilutions was generated. The slope (m), cycle threshold (C_t_) value of undiluted mengovirus, and C_t_ values of the samples were used to calculate the recovery efficiency (RE), according to the following formula:

RE (%) = 10^(ΔCt/m)^ × 100

Serial decimal dilutions of plasmids containing target viral genomes were used to construct standard curves for each virus quantification. The plotting of Ct values obtained for each dilution against log_10_ concentration (log_10_ genome copies/L of wastewater) enabled the determination of virus concentration based on the cycle threshold (C_t_) value of each sample.

#### 2.3.2. Infectivity Assay

As the presence of viral genomes in wastewater may not necessarily indicate the presence of infectious virus particles, we used a culturable virus (mengovirus) to evaluate the impact of biofiltration not only on virus quantification through qPCR, but also on virus ability to infect cells in culture, through an infectivity assay.

For such purpose, timely samples were collected during the biofiltration experiment: before the (T_0h_) and after 24 h (T_24h_), 48 h (T_48h_), 72 h (T_72h_), and 1 week (T_192h_) of treatment with clams. Each sample was filtered by a 0.2 μm cellulose membrane before inoculation on confluent monolayers of HeLa cells (ATCC^®^ CCL-2), in a 25 cm^2^ flask. Inoculated cells were incubated at 37 °C in a 5%CO_2_ atmosphere and daily observed on an inverted microscope for cytopathic effect (CPE) evaluation. Further, 0.5 mL of culture supernatant aliquot was daily collected from each flask and submitted to nucleic acid extraction and amplification of the mengovirus genome by the previously described qRT-PCR protocol.

## 3. Results and Discussion

### 3.1. Virus Removal from MWTP

The municipal wastewater treatment plant (MWTP) presents conventional treatments (biological and physical) that cannot treat the chemical and biological emerging contaminants. Some pharmaceutical and personal care products pass through the MWTP without suffering any reduction in their concentration [31]. Some enteric pathogens, such as viruses and bacteria, have also been followed at the MWTP in different countries [4,32,33,34]. One of the most common groups of detected viruses at the MW is the norovirus (genogroups I and II) and recently the SARS-CoV-2 [34,35]. In this work, influent samples used for the experimental procedure revealed the presence of JC virus, norovirus (genogroups I and II), and hepatitis A virus (HAV), while effluent samples only revealed the presence of JC virus and norovirus (both genogroups) genomes.

**Figure 3:**
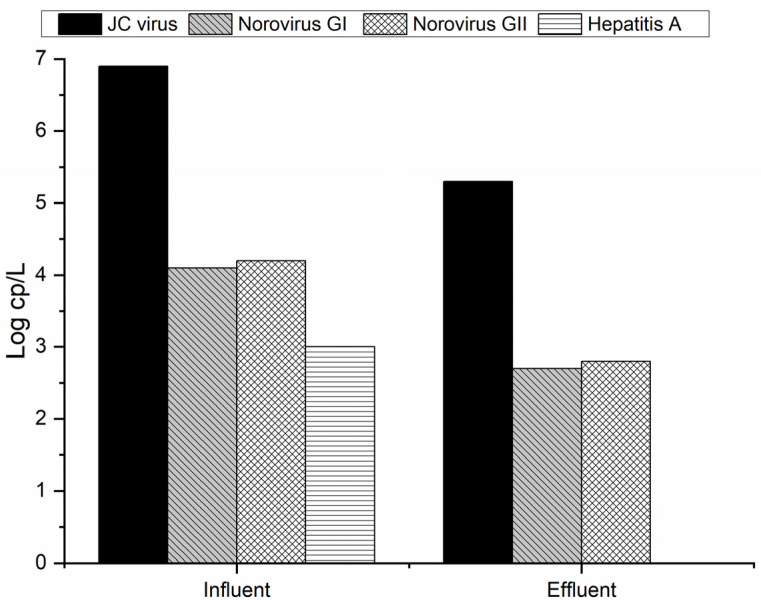
Virus concentration at the influent and effluent of the MWTP.

The analysis of the spiked process control virus (mengovirus) resulted in viral recovery efficiency values of 4.2% to 6.2%, validating all further PCR results. Each target virus was quantified by qPCR, and the obtained results are presented in Figure 3.

As can be seen in Figure 3, the JC virus is the one with the higher concentration in municipal wastewater (MW), with a value of almost 7 log cp/L. Qiu et al. [33] analyzed the UV disinfection for two different MWTPs, and the JC virus concentration at the influent of both MWTPs was about 4 log cp/L. In this sense, it is possible to see that the concentration of this virus at the influent is higher compared to the literature values. Besides this, an overall reduction in the viruses can be seen along the MWTP. However, the complete removal of JC virus and norovirus GI and GII does not occur. This is a potential hazard for human health and for those organisms in contact with such effluent when discharged to the natural water courses. During the treatment by activated sludge, it was possible to see a reduction of about 1 log cp/L for the JC virus and norovirus (GI and GII). Qiu et al. [33] for two different MWTPs equipped with UV lamps as a tertiary treatment for disinfection were not able to remove any concentration log of the previously referred virus. Despite the presence of HAV observed in the influent samples at around 3 log cp/L, it became undetectable in the effluent sample. This means that the MWTP was efficient in removing this virus. Since the structure of HAV is similar to that of norovirus, and we can therefore assume that its resistance to treatment will be identical, the efficiency in eliminating HAV should be related to its lower concentration in MW, near to the limit of detection of the used protocol (2.6 log copies/L). Prado et al. [32] analyzed the largest MWTP of Rio Janeiro (Brazil) and verified that activated sludge was able to remove the detected HAV (58% of the raw samples) from the effluent. On the other hand, in other studies, it was possible to see the resistance of the hepatitis A virus to conventional wastewater treatment since the concentration only suffered a small decrease at the same log during the biological treatment [36,37].

### 3.2. Biofiltration Through C. fluminea for Virus Removal

#### 3.2.1. Effluent MWTP

**Figure 4:**
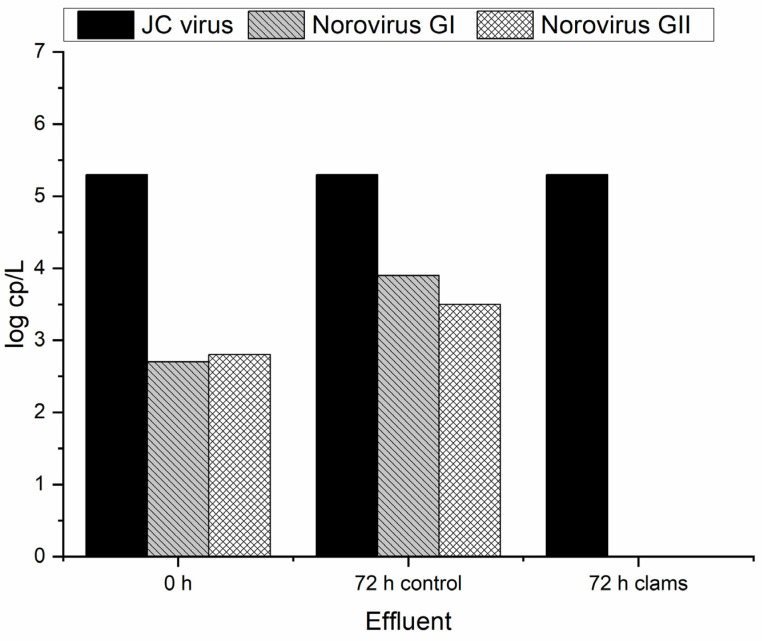
Virus concentration at the MWTP effluent after 72 h with and without clams (control).

The conventional MWTP cannot face the chemicals emerging contaminants reaching the water courses such as surface and groundwater [31,38]. Moreover, as previously presented, the great part of the virus also passes through the MWTP without suffering significant reduction. In this sense, it is important to consider new and suitable alternatives for the removal of viruses from effluents. Biofiltration through the invasive freshwater clam is an interesting solution since it can work as a pest management approach as well. In this sense, the invasive clams *C. fluminea* were placed in contact with secondary treated urban wastewater for the virus removal. The identification and quantification of detected viruses in wastewater samples are summarized in Table 2. Figure 4 reveals the virus removal through biofiltration after 72 h of effluent contact with clams. The initial sample of real wastewater corresponds to the 0 h, while the 72 h control and 72 h clams indicate the sample without and with clams, respectively. The biofiltration experiments were made in duplicate, and the standard deviation between replicates was about 2%. The data were analyzed statistically, but as the results were similar between replicates, no significant differences were found. In this way, the results can be considered reproducible, and the bivalves effectively removed the virus.

As can be seen in Figure 4, the clams were able to remove the initially present norovirus (GI and GII) in 72 h. No mortality was observed in the clams during this period of exposure. As already observed (Figure 3), activated sludge used in MWTP proved to be able to decrease the norovirus concentration by about 1 log cp/L. Moreover, it is possible to see that the concentration of norovirus GI and GII at the control spot does not significantly differ after 72 h, which proves that without any treatment this virus can persist, causing more environmental and health concerns. In this sense, it is important to apply alternative and suitable treatment since the activated sludge can only slightly decrease viral concentration (Figure 3). According to Lizasoain et al. [39], the activated sludge does not promote statistically significant differences regarding the concentration of rotavirus, human astrovirus, norovirus GII, and human adenovirus compared to the influent. On the other hand, Plaza-Garrido et al. [4] analyzed different municipal wastewater treatment plants for the norovirus GI and GII disinfection with different biological treatments, such as activated sludge, aerated lagoons, bio-disks, and constructed wetlands, among others. The activated sludge was able to remove both viruses from the influent [4]. This proves that besides the applied treatment, it is important to consider operational parameters like the residence time, dissolved oxygen, and sludge age, as well as biological characterization, initial virus concentration, and their seasonal patterns [4]. Another important feature regarding enteric virus removal was bio-disk usage since it presented the worst results compared with other biological treatment systems [4,40]. Therefore, the presence of alternative solutions for the removal of such viruses should be considered [15]. The results obtained with *C. fluminea* for the abatement of the virus show that this can be a good solution since the same clams can be reused during different cycles of treatment without any additional costs. Gomes et al. [16] analyzed the performance of these clams on *E. coli* removal during different cycles of reuse, with constant rates of removal along the cycles.

In the oldest studies that are still having importance and which are mentioned in most of the recent studies, Silverman et al. [41] used three different species of bivalves and compared their removal ability to reduce *E. coli* from artificial pond water. Using the standardization based on the dry tissue weight to provide an acceptable normalization for the comparison of the bivalve’s clearance rate, it was observed that the highest clearance rate was for *Dreissena polymorpha* (zebra mussel) species, following *C. fluminea* (Asian clam) and *Carunculina texasensis*. However, *C. fluminea* presents higher dry tissue weight and reduced gill and cirris than *D. polymorpha*, indicating that the ability to retain the bacteria is related to the architecture of the cirri on the lateral frontal cells of the gill. Further studies by the same authors [42] considered the surface area of the gill in the clearance rate values. Comparing six native rivers, three native ponds, and two invasive species, they observed a strong direct relationship between the cirral/gill surface area and the clearance rate and good efficiency of invasive species, such as zebra mussels and Asian clams, in removing *E. coli*. To understand where the *E. coli* are retained in the mussel structure, Bucci et al. [43] used simple and δ13C-labeled bacteria to feed *C. fluminea* and *Elliptio complanate*. It was observed that the bacteria are more concentrated in the gill than in the stomach of the bivalves, validating the hypothesis that *E. coli* may be aggregated on gill tissue during sorting and assimilation processes before going to the stomach. These results can indicate that the virus may also be retained by the gills and bioprocessed in the stomach of bivalves. The mechanism of removal will be assessed in further study.

Mezzanotte et al. [44] used treated effluent from a municipal wastewater treatment plant to analyze the bacterial and viral removal capacity of zebra mussels. After spiking poliovirus and rotavirus in the samples, the experiment showed that bivalves can remove enteric viruses. Still in the virus removal field, Faust et al. [22] studied the effect of Asian clams *C. fluminea* on avian influenza viruses (LPAI and H5N1 HPAI). The experiments were carried out using clams to study the removal of spiked viruses from the water. Moreover, the water and mussel tissue’s infectivity after the experiment was assessed by inoculating model indicator organisms (captive-bred wood ducks). The group of organisms inoculated with the filtered water and fed with these bivalves did not present any morbidity or mortality, unlike when exposed to the viral inoculum and the infected water not filtered by the bivalves. The results clearly show that the avian influenza viruses in water can be removed, and the infectivity is reduced by *C. fluminea*. In this sense, it is possible to conclude that besides the virus removal, the presence of clams can also decrease infectivity.

Regarding the JC virus, it is present at an elevated initial concentration, and no significant changes are observed during the biofiltration procedure. After 1 week, the concentration slightly decreased (~1 log cp/L) when in contact with the *C. fluminea*. This higher resistance of the JC virus may be related to the circular dsDNA genome, making it more chemically stable and resistant to damage compared with RNA viruses [45].

#### 3.2.2. Influent MWTP

**Figure 5:**
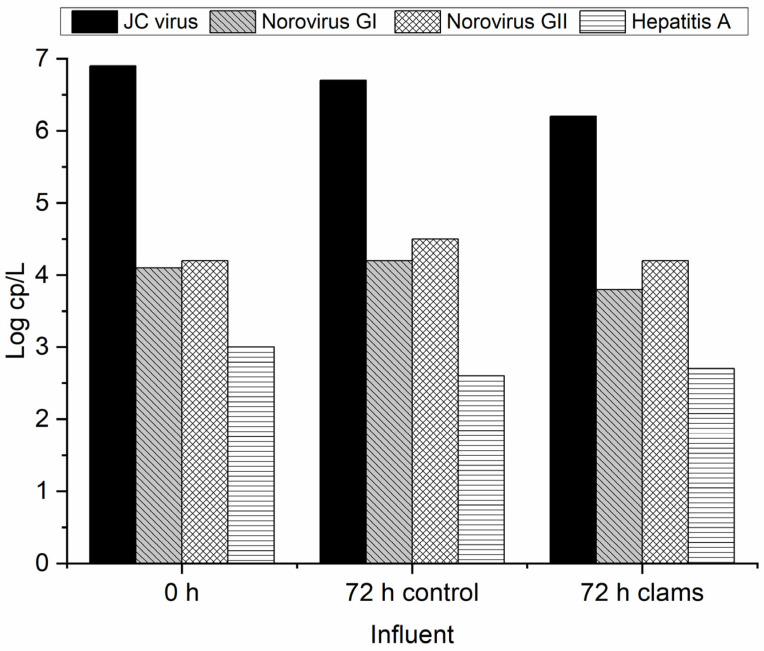
Virus concentration at the MWTP influent after 72 h with and without clams.

As previously revealed, the potential of clams for norovirus removal from municipal wastewater after secondary treatment is clear. However, to define the best location for biofiltration at the municipal wastewater treatment plants, the effect of clams in the influent of MWTP was also evaluated. Typically, this kind of influent has a great amount of organic matter, dissolved and suspended solids, which need an initial primary treatment to remove the biggest particles. In this study, the influent before this primary treatment was considered. Figure 5 presents the virus removal through biofiltration after 72 h of influent contact with clams.

Analyzing Figure 5, it can be observed the presence of HAV, JC virus, and both noroviruses after 72 h of biofiltration contact. During the experiment, a very small decrease in HAV between the blank control and biofilter is noted, indicating that the presence of bivalves does not affect the concentration of this virus in this type of sample. Contrary to the MWTP, which was able to remove the HAV after the biological treatment but not the other virus. However, using the biofilters, a slight reduction in norovirus I and JC virus concentration was observed, confirming the ability of the bivalves to remove some of the present viruses. This smaller capability of biofiltration to remove viruses from the influent wastewater can be related to the presence of higher loads of biological and chemical contaminants. Moreover, a suspended solid that passes the sieve can also affect the biofiltration performance. Some studies suggest that the limit size for particle retention by *C. fluminea* is between 20 and 50 μm [46,47]. Ismail et al. [23] used two different systems (batch and field-based flow-through) and a native species, *Anodonta californiensis*, to study the influence of initial concentrations of bacteria in the clearance rate. However, the relationship between the parameters was not linear, due to the turbidity of the lake water samples used in the batch system. Later using a native and an invasive species, *A. californiensis* and *C. fluminea* in laboratory and situ field studies, Ismail et al. showed that parameters such as bivalve type and length are important to be considered when bivalves are used as a biofilter [24]. However, in this work, the normal length size of clams used in these experiments was the same for influent and effluent conditions within the range of 25 ± 5 mm. Moreover, compared with the literature, the low performance observed in the present study was related to high organic matter content and solids fraction, which inhibit the biofiltration. In fact, Ferreira et al. [48] and Gomes et al. [25] for different wastewaters proved that the higher concentration of organic matter and solids reduces the efficiency of clam biofiltration. In this sense, during the application of the biofiltration system, it should be considered an effluent or the influent after the sedimentation/coagulation process to remove the large particles. Table 1 summarizes the results obtained for virus detection and quantification for the blank control and biofiltration using influent and effluent wastewater.

#### 3.2.3. Mengovirus Spiked Effluent Biofiltration

When using PCR techniques to evaluate the efficacy of wastewater treatments in removing viral agents, a question that often arises is the potential absence of correlation between the presence of viral genomes and infectious viruses (the presence of a viral genome may not indicate the presence of a complete viral particle, thus, the presence of infectious viruses). To clarify this relationship, we took advantage of the spiking of effluent samples with mengovirus and analyzed the presence of the viral genome and infectious virus through the biofiltration experiment. The viral genome was analyzed by qRT-PCR. The presence of infectious virus was possible due to the ability of mengovirus to grow in HeLa cells, resulting in CPE visible through inverted microscopic observation. Besides CPE observation, cell supernatant was harvested at 24 h, 48 h, and 192 h after inoculation to confirm the presence of mengovirus through real-time qRT-PCR.

This test further allows us to understand if, besides the low or very reduced amount of virus genome in the samples, the virus was completely removed or even viable as an infectious agent.

**Figure 6:**
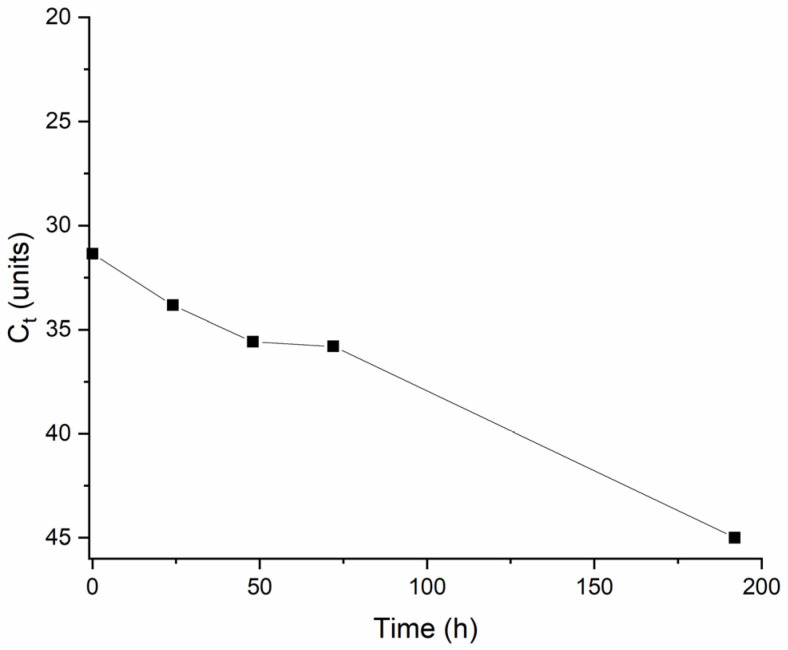
qRT-PCR C_t_ value of mengovirus in the effluent sample during biofiltration period.

An aliquot was withdrawn from the effluent sample spiked with mengovirus before being put in contact with clams, to set the baseline and confirm the viability of mengovirus (T_0_). During biofiltration treatment, an aliquot was withdrawn at 24 h (T_24_), 48 h (T_48_), 72 h (T_72_), and 1 week (T_192_). Each aliquot was inoculated in HeLa cells, with CPE and qRT-PCR for mengovirus confirmation performed at 1, 2, and 7 days after each inoculation. The quantity of mengovirus in the effluent during the treatment with clams could be inferred from the C_t_ value (indirectly proportional) obtained by qRT-PCR and is presented in Figure 6.

Analyzing Figure 6, it can be observed a reduction in mengovirus concentration through biofiltration until it became undetectable after one week of treatment (192 h), confirming the efficacy of treatment in removing the virus spiked in the effluent sample. This result validates the previous one obtained for other viruses, as would be expected since mengovirus is also a non-enveloped virus.

**Figure 7:**
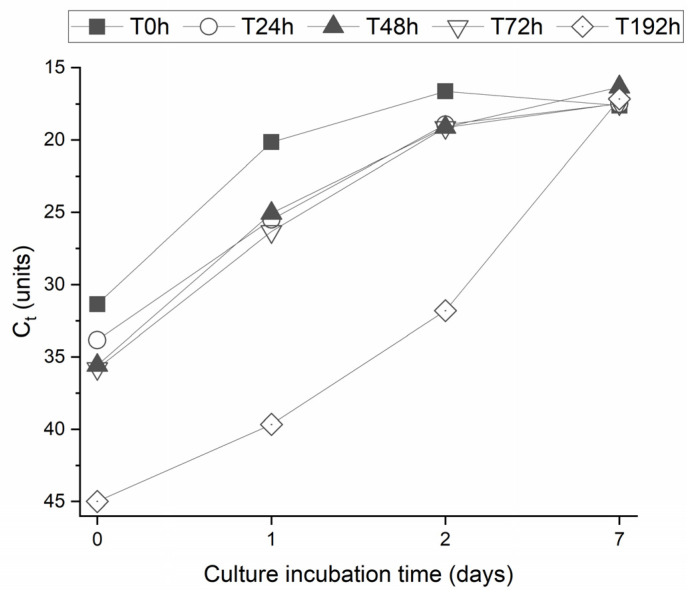
Variation in qRT-PCR C_t_ values of mengovirus through culture incubation (0, 1, 2, and 7 days) for each biofiltration period (T_0_, T_24_, T_48_, T_72_, and T_192_).

The inoculation of treated effluent in HeLa cells resulted in CPE after 48 h of incubation for T_0_, T_24_, T_48_, and T_72_, and after 72 h for T_192h_, indicating a lower amount of infectious particles in this later sample. The supernatant of each culture was submitted to qRT-PCR for mengovirus detection to confirm the origin of CPE. All harvested supernatants revealed a detectable result on qRT-PCR, confirming that CPE was induced by mengovirus replication. Figure 7 represents C_t_ values for each of the harvested samples during the 7-day culture incubation period.

As the C_t_ value on qRT-PCR inversely correlates to virus concentration, the obtained results reveal an increasing virus concentration in culture through time, as would be expected for an infectious virus inoculated on susceptible cells. These results confirm the presence of infectious virus in the inoculated samples, i.e., treated effluent samples. Treated effluent samples were evaluated by qRT-PCR before inoculation in culture, and detectable qRT-PCR results were obtained for T_0_, T_24_, T_48_, and T_72_. Thus, our results demonstrate a correlation between the presence of viral genome and infectious viruses in wastewater samples subjected to biofiltration and support the use of PCR techniques to assess the presence of infectious viruses in this type of sample and, therefore, to evaluate its microbiological safety.

Furthermore, despite spiked effluent submitted to one-week biofiltration rendering an initial undetectable qRT-PCR result, a CPE was observed after 72 h incubation on culture cells, and a positive qRT-PCR was obtained on harvested culture supernatants at 1, 2, and 7 days of incubation, confirming the presence of infectious virus particles in the inoculated sample. This result could be expected for samples with a very low number of viruses, which, despite not being enough to be detected by PCR, may be detected through culture once it enables virus replication through time until it reaches a detectable result. Regarding the Mengo virus results, the assessment was made in duplicate, and the standard deviation was about 5%. However, the results proved the virus’s viability after its removal, considering different incubation times.

## 4. Conclusions

This study analyzes virus presence along the different stages of municipal wastewater treatment plants (MWTP). The JC virus, norovirus (genogroups I and II), and hepatitis A virus were identified as the influent of MWTP. Moreover, the biological treatment of MWTP was able to remove the hepatitis A virus below the detection level, while the other identified viruses did not have any reduction. To overcome this MWTP drawback, the invasive species *Corbicula fluminea* proved that it can be a suitable solution since it can biofilter some viruses, such as norovirus GI and GII. Therefore, the application of this bivalve to improve wastewater treatment regarding viruses can work as a pest management approach with the correct biofilter design. However, this treatment should encompass an oxidative process since the complete removal of the virus below the detection level of PCR quantification may not represent complete disinfection, as evidenced by infectivity assays. Furthermore, the results from infectivity assays strongly support the use of PCR for evaluating the microbiological quality of effluents. Also, to guarantee complete disinfection, the JC virus concentration can be used as a reference indication of wastewater quality since it is the most resistant virus.

## Figures and Tables

**Table 1:** Nucleotide sequence of primers and probes used on real-time PCR protocols for detection and quantification of JC virus, norovirus genogroups I and II, hepatitis A virus, and mengovirus.

Target Virus	Target Region or Group	Nucleotide Sequence (5′-3′)		Annealing Temperature	Reference
JC virus	AgT	Forward primer	AGT GTT GGG ATC CTG TGT TTT CA	60 °C	[27]
		Reverse primer	GTG GGA TGA AGA CCT GTT TTG C		
		Probe	CAT CAC TGG CAA ACA T		
	NCCR	Forward primer	GGA GCC CTG GCT GCA T		
		Reverse primer	TGT GAT TAA GGA CTA TGG GAG G		
		Probe	CTG GCA GTT ATA GTG AAA CC		
Norovirus	Genogroup I	Forward primer	CGC TGG ATG CGN TTC CAT	60 °C	[28]
		Reverse primer	CCT TAG ACG CCA TCA TCA TTT AC		
		Probe	TGG ACA GGA GAY CGC RAT		
	Genogroup II	Forward primer	ATG TTC AGR TGG ATG AGR TTC TCW GA	60 °C	
		Reverse primer	TCG ACG CCA TCT TCA TTC ACA		
		Probe	AGC ACG TGG GAG GGC GAT CG		
Hepatitis A virus		Forward primer	TCA CCG CCG TTT GCC TAG	60 °C	[29]
		Reverse primer	GGA GAG CCC TGG AAG AAA G		
		Probe	CCT GAA CCT GCA GGA ATT AA		
Mengovirus		Forward primer	GCG GGT CCT GCC GAA AGT	60 °C	[30]
		Reverse primer	GAA GTA ACA TAT AGA CAG ACG CAC AC		
		Probe	ATC ACA TTA CTG GCC GAA GC		

**Table 2:** Viruses’ identification and quantification for blank control and biofiltration procedures using influent and effluent wastewater (where ND = not detected).

	Time (h)	JC Virus		Norovirus GI		Norovirus GII		HAV	
		cp/L	log cp/L	cp/L	log cp/L	cp/L	log cp/L	cp/L	log cp/L
Effluent control	0	1.89 × 10^5^	5.3	4.85 × 10^2^	2.7	6.04 × 10^2^	2.8	ND	
	72	2.11 × 10^5^	5.3	8.14 × 10^3^	3.9	2.98 × 10^3^	3.5	ND	
Effluent with clams	0	1.89 × 10^5^	5.3	4.85 × 10^2^	2.7	6.04 × 10^2^	2.8	ND	
	72	2.12 × 10^5^	5.3	ND		ND		ND	
Influent Control	0	7.55 × 10^6^	6.9	1.25 × 10^4^	4.1	1.61 × 10^4^	4.2	9.52 × 10^2^	3.0
	72	4.53 × 10^6^	6.7	1.50 × 10^4^	4.2	3.48 × 10^4^	4.5	4.35 × 10^2^	2.6
Influent with clams	0	7.55 × 10^6^	6.9	1.25 × 10^4^	4.1	1.61 × 10^4^	4.2	9.52 × 10^2^	3.0
	72	1.60 × 10^6^	6.2	6.41 × 10^3^	3.8	1.74 × 10^4^	4.2	5.53 × 10^2^	2.7

## Data Availability

No data is available.
